# Marked Hypervitaminemia B12 Associated With Previously Unrecognized Alcohol-Related Cirrhosis Revealed During Alcohol Withdrawal

**DOI:** 10.14740/jmc5323

**Published:** 2026-07-01

**Authors:** Abrar-Ahmad Zulfiqar

**Affiliations:** Geriatrics Unit, Hopital des Hautes Falaises, 76400 Fecamp, France. Email: abzulfiqar@gmail.com

**Keywords:** Vitamin B12, Hypervitaminemia B12, Liver disease, Alcohol-related cirrhosis

## Abstract

Vitamin B12 deficiency is commonly investigated in clinical practice, particularly in patients with anemia, neurological disorders, or malnutrition. In contrast, elevated serum vitamin B12 levels are less frequently discussed and may be overlooked by clinicians. However, hypervitaminemia B12 has been associated with several underlying conditions, including liver diseases, hematological malignancies, solid tumors, and chronic kidney disease. Liver injury may lead to increased circulating levels of vitamin B12 due to hepatocellular release and altered metabolism of cobalamin-binding proteins. We report the case of a 44-year-old man admitted for planned alcohol withdrawal after a 10-year history of chronic alcohol consumption estimated at 80–100 g of ethanol per day. Physical examination revealed moderate facial couperosis, a sharp inferior liver edge on palpation, and a few spider angiomas without ascites or signs of hepatic encephalopathy. Laboratory investigations showed macrocytosis without anemia, mild thrombocytopenia, moderate hepatic cytolysis, and markedly elevated gamma-glutamyl transferase levels. A significant hypervitaminemia B12 was identified with a serum concentration of 1,124 pg/mL. Additional findings included mild hyperferritinemia, albumin at 32 g/L, and prothrombin time at 75%. Alpha-fetoprotein levels were within the normal range. Abdominal ultrasound demonstrated morphological features consistent with liver cirrhosis without focal hepatic lesions. The patient was classified as Child-Pugh class A, and the FIB-4 score was calculated at 6.2, suggesting advanced liver fibrosis. This case highlights that elevated serum vitamin B12 may represent an important biological marker of underlying liver disease, particularly alcohol-related cirrhosis. Recognition of hypervitaminemia B12 in patients with chronic alcohol consumption should prompt further evaluation for liver disease and may contribute to earlier detection of chronic hepatopathies.

## Introduction

Vitamin B12, also known as cobalamin, is an essential water-soluble vitamin involved in several fundamental biological processes, including the synthesis of deoxyribonucleic acid (DNA), the maturation of red blood cells, and the functioning of the nervous system. It acts as an enzymatic cofactor in the metabolism of homocysteine and methylmalonyl-CoA, two metabolic pathways essential for cellular function. In clinical practice, vitamin B12 status is generally assessed as part of screening for deficiency, particularly in patients with macrocytic anemia, neurological disorders, or malnutrition.

Conversely, hypervitaminemia B12 is a biological abnormality that has been much less studied and is often underestimated by clinicians. However, several studies have shown that high concentrations of vitamin B12 may be associated with various underlying conditions, some of which can be severe. The main causes described include liver disease, certain malignant blood disorders, solid cancers, and chronic kidney disease [1, 2].

The liver plays a central role in vitamin B12 metabolism, as it is the main storage site for cobalamin in the body. Under physiological conditions, vitamin B12 circulates in the plasma bound to two main transport proteins: transcobalamin and haptocorrin. Transcobalamin transports the biologically active fraction to the tissues, while haptocorrin transports the majority of circulating vitamin B12 but corresponds to a biologically inactive fraction [3].

In liver disease, several mechanisms can lead to elevated serum vitamin B12 concentrations. The destruction of hepatocytes can cause intracellular cobalamin to be released into the bloodstream. In addition, alterations in the metabolism of vitamin B12 transport proteins can contribute to increased plasma concentrations [4].

Several studies have shown that hypervitaminemia B12 is frequently observed in chronic liver diseases, particularly in acute hepatitis, alcoholic steatohepatitis, and cirrhosis. In these situations, elevated vitamin B12 levels may reflect hepatocellular destruction and constitute an indirect biological marker of liver dysfunction [5].

We report here the case of a patient hospitalized for alcohol withdrawal whose initial blood tests revealed marked hypervitaminemia B12, leading to a diagnosis of compensated alcoholic cirrhosis.

## Case Report

A 44-year-old man was admitted to an internal medicine ward for planned alcohol withdrawal. The patient’s main medical history was chronic alcoholism for approximately 10 years, with an estimated daily consumption of between 80 and 100 g of ethanol. He reported no previous withdrawal and was not taking any regular medication.

On admission, the patient’s general condition was stable and his hemodynamic parameters were normal.

Clinical examination revealed moderate facial rosacea associated with the presence of a few stellate angiomas on the chest. Abdominal palpation revealed a palpable lower edge of the liver in the right hypochondrium, described as sharp.

No signs of advanced portal hypertension were observed. There was no clinically detectable abdominal collateral venous circulation or ascites. The neurological examination was normal and the patient showed no signs of hepatic encephalopathy.

A complete blood count was performed on admission (Table 1). The blood count showed normal hemoglobin levels with no anemia. Macrocytosis was observed with a mean corpuscular volume of 104 fL. Moderate thrombocytopenia was also found.

**Table 1:** Biological Findings

Parameter	Result	Normal range
Hemoglobin	14.6 g/dL	13–17
Mean corpuscular volume	104 fL	80–98
Platelets	118 × 10^9^/L	150–400
Aspartate aminotransferase	132 IU/L	< 40
Alanine aminotransferase	98 IU/L	< 45
Gamma-glutamyl transferase	420 IU/L	< 60
Bilirubin	26 µmol/L	< 20
Albumin	32 g/L	35–50
Total protein	75 g/L	70–100
Ferritin	640 µg/L	30–300
Vitamin B12	1,124 pg/mL	200–900
Alpha-fetoprotein	3.2 ng/mL	< 10

Liver function tests revealed moderate cytolysis with aspartate aminotransferase and alanine aminotransferase levels approximately three times higher than normal. A marked elevation in gamma-glutamyl transferase was observed.

The blood tests also revealed moderate hyperferritinemia.

The most notable biological abnormality is the presence of significant hypervitaminemia B12, with a measured level of 1,124 pg/mL. The unexpected discovery of marked hypervitaminemia B12 prompted further evaluation for underlying conditions known to be associated with elevated cobalamin levels, particularly chronic liver disease, hematological disorders, and solid malignancies. In the present case, the coexistence of macrocytosis, thrombocytopenia, elevated gamma-glutamyl transferase, and mild impairment of liver synthetic function strongly suggested chronic alcohol-related liver injury.

Consequently, additional hepatic investigations were performed, including abdominal ultrasonography and non-invasive fibrosis assessment using the FIB-4 score, both of which supported the diagnosis of compensated cirrhosis. Alpha-fetoprotein levels remained within the normal range, and no focal hepatic lesion was identified on imaging.

During hospitalization, the patient underwent alcohol withdrawal management associated with vitamin supplementation, nutritional counseling, and hepatological follow-up recommendations. Long-term alcohol abstinence was strongly encouraged, and regular monitoring for complications of chronic liver disease, including hepatocellular carcinoma surveillance and biological follow-up, was recommended after discharge.

An abdominal ultrasound shows a dysmorphic liver consistent with cirrhosis.

The Child-Pugh score is 6 (class A) and the FIB-4 score is 6.2, consistent with advanced fibrosis.

## Discussion

Hypervitaminemia B12 is a biological abnormality that is still insufficiently recognized in clinical practice. Unlike vitamin B12 deficiency, which is frequently screened for, elevated serum cobalamin concentrations are often overlooked. However, several studies have shown that this abnormality can be an indirect marker of underlying pathologies that are sometimes severe [1].

The liver is the main reservoir of vitamin B12 in the body. Under physiological conditions, most vitamin B12 is stored in hepatocytes. In liver disease, the destruction of hepatocytes can lead to the release of cobalamin into the bloodstream, resulting in elevated serum concentrations [4].

Alterations in the metabolism of vitamin B12 transport proteins also play an important role. Haptocorrin, produced mainly by granulocytes and certain liver cells, transports most of the circulating vitamin B12. In certain diseases, particularly liver disease and certain cancers, an increase in circulating haptocorrin can lead to elevated serum vitamin B12 concentrations [3].

In chronic liver diseases, elevated vitamin B12 levels have been reported in several situations, including acute hepatitis, alcoholic steatohepatitis, and cirrhosis [5].

Some studies have also shown that vitamin B12 concentrations may be correlated with the severity of liver disease. Particularly high levels have been observed in patients with advanced cirrhosis or severe hepatocellular insufficiency [6].

In addition, high vitamin B12 concentrations have been reported in certain malignant blood disorders and solid cancers, particularly due to increased production of transport proteins by tumor cells [2].

More recently, several epidemiological studies have shown that high vitamin B12 concentrations may be associated with increased all-cause mortality. In a large population cohort, Flores-Guerrero et al showed that high vitamin B12 concentrations were associated with an increased risk of mortality [7].

More recent data also suggest that hypervitaminemia B12 could be a biological marker of metabolic dysfunction or systemic inflammation in certain chronic diseases [8].

In the case presented, hypervitaminemia B12 is observed in a clinical context consistent with chronic alcoholic liver disease. The associated biological abnormalities, including macrocytosis, thrombocytopenia, and significantly elevated gamma-glutamyl transferase, are classic biological markers of chronic alcohol consumption. The high FIB-4 score reinforces the hypothesis of advanced liver fibrosis.

Thus, the hypervitaminemia B12 observed in this case can be interpreted as an indirect biological marker of chronic liver disease, which may guide diagnosis in patients with excessive alcohol consumption.

The elevated vitamin B12 level observed in this patient is likely multifactorial. The liver constitutes the main storage site for cobalamin, and chronic hepatocellular injury may induce the release of stored vitamin B12 into the circulation. In addition, liver dysfunction may alter the metabolism and clearance of vitamin B12-binding proteins, particularly haptocorrin, leading to increased circulating serum vitamin B12 concentrations.

Chronic alcohol-related liver disease may therefore produce hypervitaminemia B12 not because of excessive intake, but as a consequence of hepatocellular damage and impaired hepatic metabolism. In this context, elevated vitamin B12 may represent an indirect marker of liver dysfunction and fibrosis severity.

Observations reported in the literature confirm that hypervitaminemia B12 is frequently observed in chronic liver diseases, particularly in alcoholic cirrhosis. In a study of patients with liver disease, Sugihara et al showed that serum vitamin B12 concentrations were significantly higher in patients with cirrhosis than in patients with less advanced liver disease [5]. These results suggest that elevated vitamin B12 levels may reflect the extent of hepatocellular destruction and loss of liver function.

Several authors have also suggested that hypervitaminemia B12 may be associated with the severity of certain liver diseases. In some series, particularly high concentrations have been observed in patients with advanced hepatocellular insufficiency or decompensated cirrhosis [6]. In these situations, elevated vitamin B12 levels may be related to the massive release of cobalamin stored in hepatocytes and to an alteration in the metabolism of vitamin B12 transport proteins.

Recent studies have also suggested that hypervitaminemia B12 could be a biological marker of poor prognosis in certain chronic diseases. In a large population cohort, Flores-Guerrero et al showed that high plasma vitamin B12 concentrations were associated with a significant increase in all-cause mortality [7]. These results suggest that hypervitaminemia B12 could reflect the existence of an underlying systemic disease or chronic inflammatory state.

In our observation, hypervitaminemia B12 was discovered during a biological assessment carried out during alcohol withdrawal. This clinical context is particularly interesting because it illustrates how an apparently isolated biological abnormality can lead to the discovery of underlying chronic liver disease. The association of hypervitaminemia B12 with other biological abnormalities characteristic of chronic alcoholism, such as macrocytosis, thrombocytopenia, and significantly elevated gamma-glutamyl transferase, should alert the clinician and prompt investigation for liver damage.

This case highlights the importance of careful interpretation of biological abnormalities in clinical practice. Hypervitaminemia B12 should not be considered a simple benign biological abnormality but should instead prompt investigation for underlying pathology, particularly chronic liver disease, malignant hematological disease, or solid cancer.

Hypervitaminemia B12 is increasingly recognized as an underestimated biological abnormality that should not be dismissed as incidental. Zulfiqar and colleagues reported that elevated serum vitamin B12 levels are frequently associated with significant underlying conditions, including solid neoplasms, hematological disorders, and chronic liver disease, and suggested that this finding should prompt a structured diagnostic evaluation [9, 10].

This report has some limitations inherent to its design as a single case report. First, causality between hypervitaminemia B12 and liver disease cannot be formally established. Elevated vitamin B12 concentrations may occur in other clinical situations, including hematological malignancies, solid tumors, inflammatory conditions, and renal dysfunction. Although no clinical or biological findings suggested these alternative diagnoses in the present patient, exhaustive exclusion cannot be guaranteed. Second, longitudinal follow-up was not available, and serial vitamin B12 measurements after alcohol withdrawal were not performed. Consequently, the impact of alcohol abstinence and hepatic evolution on serum vitamin B12 concentrations could not be assessed. Further prospective studies are needed to clarify the clinical significance and prognostic value of hypervitaminemia B12 in chronic liver disease.

### Conclusions

This clinical case illustrates the discovery of compensated alcoholic cirrhosis in a patient hospitalized for alcohol withdrawal whose initial laboratory tests revealed marked hypervitaminemia B12.

Although vitamin B12 is most often studied in situations of deficiency, its elevation in serum may constitute a clinically relevant biological association that should prompt further evaluation for underlying liver disease.

In patients with chronic alcohol consumption, the discovery of hypervitaminemia B12 should prompt investigation for underlying liver damage.

### Learning points

Elevated vitamin B12 levels should not be considered a benign laboratory finding. Although vitamin B12 testing is usually performed to detect deficiency, markedly elevated serum levels may represent an indirect biological marker of underlying diseases, including liver disease, hematological malignancies, solid tumors, or chronic kidney disease.

Chronic liver disease, particularly alcohol-related cirrhosis, can lead to hypervitaminemia B12. This increase is mainly explained by the release of stored cobalamin from damaged hepatocytes and alterations in vitamin B12–binding proteins, reflecting hepatocellular injury.

In patients with chronic alcohol consumption, the discovery of hypervitaminemia B12 should prompt evaluation for underlying liver disease. When associated with other biological abnormalities such as macrocytosis, thrombocytopenia, and elevated gamma-glutamyl transferase, it may help identify advanced fibrosis or compensated cirrhosis at an early stage.

## Data Availability

All relevant data supporting the findings of this case report are included within the article.
